# Involvement of Ghrelin Dynamics in Stress-Induced Eating Disorder: Effects of Sex and Aging

**DOI:** 10.3390/ijms222111695

**Published:** 2021-10-28

**Authors:** Chihiro Yamada

**Affiliations:** Tsumura Kampo Research Laboratories, Tsumura & Co., Ibaraki 300-1192, Japan; yamada_chihiro@mail.tsumura.co.jp

**Keywords:** stress, appetite, ghrelin, sex differences, aging

## Abstract

Stress, a factor that affects appetite in our daily lives, enhances or suppresses appetite and changes palatability. However, so far, the mechanisms underlying the link between stress and eating have not been fully elucidated. Among the peripherally produced appetite-related peptides, ghrelin is the only orexigenic peptide, and abnormalities in the dynamics and reactivity of this peptide are involved in appetite abnormalities in various diseases and psychological states. This review presents an overview of the research results of studies evaluating the effects of various stresses on appetite. The first half of this review describes the relationship between appetite and stress, and the second half describes the relationship between the appetite-promoting peptide ghrelin and stress. The effects of sex differences and aging under stress on appetite are also described.

## 1. Overview of Stress and Feeding

Stress may be defined as the physiological process of what is perceived to be harmful to an individual. We routinely experience various types of mild to severe stress. For example, emotional impacts resulting from environmental changes and sad experiences, such as the death of a family member or a close acquaintance, and intense painful physiological impacts, such as dietary restrictions and injuries, are also defined as stress. Stress can affect biological functions, depending on its type, severity, and duration. Stressors commonly experienced in daily life affect appetite [1,2], gastrointestinal motility [3,4], and digestive function [5]. Furthermore, the physiological response may differ depending on whether the events are acute or chronic. Acute stress is considered an adaptive mechanism of the body, but chronic stress can damage various biological functions. Notably, several factors paradoxically influence the appetitive response to stressors. Mild but acute stress and intense stress, for instance, enhance the release of adrenaline and glucocorticoids [6] and reduce appetite. The persistence of this condition affects energy balance and can result in nutritional problems. Furthermore, constant exposure to stressors in daily life increases appetite and potentially causes bulimia [7,8]. Chronic stress affects gastrointestinal functions, such as motility [9], and brain functions, such as cognition, memory, and learning [10,11]. Chronic stress can affect food preferences and promote the craving and intake of palatable high-fat and energy-dense foods [12,13]. Under chronic stress, the glucose and salt susceptibility threshold is lowered and sugar intake is increased [14]. An important area of the brain activated by stress is the ventral tegmental area (VTA), or nucleus accumbens, and chronically given stressors act on the reward system to increase appetite or change the taste of food. Thus, the basic or clinical outcomes suggesting that stress increases appetite, alters palatability, and causes weight gain are beginning to increase. However, so far, the clinical evaluation of stress-reducing appetite remains limited and the elucidation of the underlying mechanism using animals has not progressed much.

Clinical trials in which participants are stressed to reduce their food intake are ethically problematic and require ingenuity. Additionally, few clinical indicators objectively evaluate appetite. The amount of consumed food, calorie intake, three-factor eating questionnaire [15], Satter eating competence inventory [16], and perceived stress scale [17] are used to measure appetite. However, under stress, there remains a debate whether the application of the former two is appropriate. In basic research, it is common to measure the amount of food consumed during a fixed time [18]. Moreover, the time to approach food (i.e., latency to eat), such as graham cracker crumbs, and the amount of food eaten first (i.e., first meal amount) may be evaluated as motivation for eating [19,20]. The feeding behavior of mice has recently been automatically measured, and meal patterns, such as the number of bouts, their intervals, and meal size have been evaluated [21,22]. In addition to glucocorticoid levels in the blood, food intake has become the most associated event that occurs under stress. Anorexia and bulimia are believed to be caused by multiple known and unknown biological factors. So far, the advancements in brain analysis through neuroimaging have revealed some dysfunctions common to each symptom [23]. Furthermore, endocrinological approaches have been used to elucidate the potential role of dysfunction in several neuropsychiatric and appetite-related peptides. Such approaches are an important tool for determining the detailed mechanisms of anorexia and overeating. However, the detailed mechanism of an eating abnormality during stress has not been elucidated in depth.

This review focuses on the suppressive effect of stress on appetite: (1) Typical intrinsic factors that control appetite and stress; (2) ghrelin dysfunction; (3) the relationship between ghrelin reactivity and sex; and (4) the effects of aging and ghrelin.

## 2. Peptides That Affect Appetite under Stress

Most appetite-related peptides produced in the digestive tract and released into the blood are appetite-suppressing hormones. Leptin, an anorexic peptide, is produced primarily by adipocytes and acts on the arcuate nucleus of the hypothalamus. Although the coexistence of elevated leptin levels with obesity is widely interpreted as evidence of “leptin resistance”, the relationship between leptin and anorexia nervosa/bulimia has not been completely elucidated. Recently, leptin was shown to control the energy balance between stress and energy metabolism [24]. The secretion of cholecystokinin (CCK), peptide YY (PYY), and glucagon-like peptide 1 (GLP-1) is controlled by sensing lipids and glucose in the digestive tract. Furthermore, the involvement of CCK and PYY under stress has been suggested [25,26,27]. Restraint stress amplified the suppressive effect of CCK on food intake [25]. Foot shock-induced stress resulted in increased CCK levels in the posterior arcuate nucleus. However, notably, stress-induced CCK depletion occurs in the ventromedial and dorsomedial hypothalamic regions [27]. The roles of PYY and GLP-1 in feeding were demonstrated using restraint [26] or water immersion stress [28]. Peripheral PYY and GLP-1 levels increased in mice immersed in water, demonstrating the possibility of a mediating part of the mechanism that reduces feeding in stress models [28]. However, no direct evidence of the role of appetite-suppressing peptides in stress feeding was revealed, and additional verification of the detailed mechanism is warranted.

Kangawa and Kojima [29] discovered ghrelin, an orexigenic hormone localized in the gastric fundus, in 1999. It is the only peripherally produced orexigenic hormone that is a unique peptide with 28 amino acids and an *n*-octanoyl group. Peripheral ghrelin production and secretion are regulated by hunger status. Ghrelin is secreted into the blood from X/A-like cells in the stomach 1–2 h before daily mealtime, thereby increasing its blood concentration; the secretion decreases by 1 h after the meal, thereby decreasing its blood concentration [30]. Thus, it may play a role in signaling and reporting hunger to the central nervous system (CNS). Ghrelin produced in the gastric mucosa acts on ghrelin receptors located at the ends of the nearby vagus nerve, which transmits these signals to the CNS. The signal via the solitary nucleus (NTS) of the medulla oblongata is an input for the arcuate nucleus of the hypothalamus and transmits an electrical signal to neuropeptide Y (NPY)/agouti-related peptide (AgRP) neurons. In addition, ghrelin promotes the production of peptides, such as NPY and AgRP, and induces appetite [31]. The action of ghrelin may also be affected by anorexic peptides, such as CCK [32]. It has been demonstrated that the ghrelin signal from the periphery is suppressed by the appetite-suppressing signal of peripherally administered CCK during the process of being transmitted to the brain [32]. Ghrelin is also present in trace amounts in the CNS and acts as a neurotransmitter [33,34]. Ghrelin administered to the brain strongly stimulates appetite. It has also been hypothesized that ghrelin released into the periphery permeates the relatively loose regions of the blood–brain barrier, such as the area postrema, and acts directly on the brain. Banks and colleagues also propose the existence of ghrelin transporters in the blood–brain barrier [35]. Thus, ghrelin may act as a neurotransmitter produced in the CNS and a signal from the periphery.

The ghrelin receptor, growth hormone secretagogue receptor 1a (GHS-R1a), is widely distributed in the body, particularly in the hypothalamus, which is the center of appetite, the stress-sensitive amygdala, and the VTA, which is associated with feeding motivation [36,37,38,39]. When subjected to repeated stress, the mechanism that stimulates the reward system becomes overactive, resulting in overeating. GHS-R activation in this region plays a crucial role in binge eating. However, the mechanism of loss of appetite due to acute stress (e.g., pain caused by illness or injury) remains largely unclear. A further challenge is that most of the links between appetite-regulating peptides and stress have been established via basic research using rodents, and there are few clinical studies. Stress susceptibility varies significantly from person to person, and overcoming the precision of peptide quantification, which is susceptible to psychological effects, may lead to further development in this field.

## 3. Changes in Ghrelin Secretion and Reactivity to Stress

Since the discovery of ghrelin, its association with various diseases has been extensively studied. Recently, it has become clear that attenuated ghrelin signaling plays a vital role in reducing food intake under stress [2,40,41,42]. To date, basic research has demonstrated that blood ghrelin levels are reduced in animal models of a disease that clinically causes anorexia and that exogenous ghrelin supply restores appetite. The administration of drugs, such as anticancer drugs and classic antidepressants, causes various stresses, such as nausea and vomiting, and loss of appetite, leading to reduced treatment compliance. These drugs also deplete ghrelin levels in the body [43,44,45].

Stress is a trigger for diseases that cause psychological abnormalities. We found that a decrease in ghrelin levels was associated with hypophagia after exposure to acute stress. Urocortin is a biological peptide that strongly binds to corticotropin-releasing factor (CRF) 1 and 2 receptors and causes stress-like symptoms. Urocortin intraventricularly administered in rats markedly reduces food intake and blood ghrelin levels [40]. Similar results were observed in mice exposed to restraint stress and immune-associated stress due to lipopolysaccharide injection [46,47]. Strong stressors are transiently loaded in these stress models and have systemic effects. Such stress causes ghrelin depletion in parallel with feeding suppression. In contrast, mice subjected to chronic social defeat and chronic isolation stress demonstrate increased peripheral ghrelin production and food intake compared with unstressed mice [48,49]. However, no weight gain commensurate with increased feeding is observed in mice subjected to chronic isolation. Conversely, in mice immersed in water [28] and those with stress due to a novel environment [42], the peripheral ghrelin level increases after the stress load, but the food intake decreases. This suggests “ghrelin resistance”, signifying the inhibition of the transmission of peripheral ghrelin signals to the CNS. Thus, the resulting change in feeding behavior depends on the type and quality of stress, and may be mediated by abnormal ghrelin dynamics.

Although there is little clinical evidence to clarify the association between stress and ghrelin, Kiessl et al. evaluated food intake and peripheral ghrelin levels before and after acute stress loading using the trier social stress test (TSST) as a stressor. Acute stress suppresses feeding and does not affect blood ghrelin levels [50]. Conversely, Rouach et al. demonstrated that TSST loading increased peripheral ghrelin levels in cortisol responders and that there was a positive association among alterations in ghrelin and cortisol levels [51]. Mckay et al. reported that ghrelin levels in participants were significantly higher after stress compared with the baseline [52]. They concluded that ghrelin is the most likely candidate driving energy intake after stress in humans. Similarly, TSST loading increases ghrelin levels to a greater extent in subjects with dietary restrictions as compared with those with no dietary restrictions, but no significant differences were detected for PYY [53]. These results were consistent with the findings of studies that reported increased peripheral ghrelin in a mouse acute stress model due to water immersion load [28] and novel environmental change load [42]. Thus, clinical trials in which acute stress loading increased peripheral ghrelin levels but reduced appetite were reproducible in several psychological stress models.

## 4. Relationship between Ghrelin Reactivity and Sex Due to Stress Loading

The main neural responses to stress are the activation of the paraventricular nucleus (PVN) of the hypothalamus and the resulting release of neuropeptides that activate the pituitary gland, CRF, and vasopressin. This activates the hypothalamo–pituitary–adrenal (HPA) axis, which represents the stress feedback mechanism [54,55]. The stress response is influenced by sex, and females are considered more vulnerable than males [56,57]. Additionally, depression and bulimia caused by stress are more prevalent in women [58,59]. Studies on rodents exposed to multiple stressors demonstrated elevated levels of corticosterone (CORT) and adrenocorticotropic hormone (ACTH) in females than in males [60,61]. Following stress, neural activity in the PVN is also high in females [62]. Compared with male rats, female rats have higher levels of arginine vasopressin (AVP) and CRF mRNA expression in the PVN, and higher levels of ACTH precursor proopiomelanocortin (POMC) mRNA in the anterior pituitary gland after induction with acute stressors [63,64]. Additionally, the restoration of ACTH and CORT levels to the baseline after acute stress in female rats is delayed, and female rats are vulnerable to negative feedback regulation of the HPA axis [61,65]. Glucocorticoid binding to target tissues, which play an essential role in negative feedback, is lower in the hypothalamus of female rats than in the hypothalamus of male rats, suggesting that the female hypothalamus has fewer corticosteroid receptors [66]. These findings indicate that the same kind of stress and similar stress load result in differences in subsequent biological reactions in males and females.

Furthermore, the sex differences in the neuroendocrine response to acute stress are partly caused by the interaction between the HPA axis and the hypothalamic–pituitary–gonadal (HPG) axis [67], which controls reproduction. However, the exact mechanism of gonadal hormones on stress response is under investigation. The activation of the HPG axis drives estrogen and androgen production in the testes and ovaries, resulting in physiological changes in the female estrous cycle. Estrogen enhances HPA axis response and promotes negative feedback, whereas estradiol treatment has the opposite effects [68,69]. Estradiol-induced increased *CRF*, *AVP*, and *POMC* gene expression causes HPA axis activation [70,71,72]. However, estrogen exerts the opposite effect on the HPA axis via two receptors. Estrogen receptor (ER) α activation indirectly causes HPA axis activation [73], whereas ERβ acts on the PVN and inhibits HPA axis activity [74,75]. Thus, the regulation of the HPA axis by the two ERs may be influenced by several factors, including stress and the reproductive cycle.

The relationship between feeding and gonadal hormones is relatively widely understood. In rodents, these hormones play important roles in energy intake to maintain homeostasis [76]. This is evident in ovariectomized animals, which have increased food intake, and orchidectomized animals, which have decreased food intake. The supply of each hormone normalizes feeding behavior [77,78]. There is also direct evidence that administering estrogen preparations reduces food intake [76]. However, the effects of gonadal hormones on feeding behavior under stress can be complex.

Studies using psychologically stressed mice have reported various findings. In one study, the mice were acclimated for ≥1 week in group housing and then individually transferred to different cages with new bedding and food. This model showed a mild stress response in male mice, with a slight decrease in food intake that recovered to the same level as the control mice within 3 h after an increase in plasma ghrelin levels [42]. Conversely, the decrease in food intake continued even 6 h after stress loading in female mice, although the peripheral ghrelin levels increased. When CORT levels were used as an index, no sex differences were noted in dynamic CORT level changes due to stress, although females showed higher levels than males [42]. This result suggests that the poststress feedback system may be delayed at a point different from the poststress glucocorticoid production process. Although this appears to be a paradoxical phenomenon, in female mice, ghrelin resistance was observed, indicating that an increase in ghrelin does not lead to feeding behavior due to stress loading.

Assessing responsiveness to exogenous ghrelin is a useful measure for determining ghrelin resistance. When acylated ghrelin is administered to normal mice, they consume more food. Furthermore, normal female mice appear more responsive than male mice to ghrelin [42]. Intraperitoneal administration of 50–500 nmol/kg of acylated ghrelin resulted in an increase in food intake, which persisted for 6 h and 24 h in female mice, but not in male mice [42,79]. Because male mice were used in most studies on ghrelin reactivity in previous reports, this is the first report on the high reactivity of female mice. After ghrelin administration, electrical stimulation of nerves from the NTS to the arcuate nucleus was higher in females than in males, and AgRP gene expression in the hypothalamus was increased. Conversely, stress loading on female mice inhibited strong neural signals post NTS induced by ghrelin administration. This implies that stress can directly affect the NTS, which is the relay point for ghrelin signals. Furthermore, it was confirmed that ERα-expressing nerves were activated in the NTS. ERα activation can inhibit the ghrelin signal. The consequences of acute psychological stress in mice may suggest a potential mechanism of stress-induced feeding suppression in women.

In our previous study, chronic isolation stress for 2 weeks significantly increased food intake in male mice [49]. This increase also resulted in an increase in peripheral ghrelin levels and the gene expression of the orexigenic peptide NPY/AgRP in the hypothalamus. Thus, binge eating in stressed male mice appears to be directly associated with increased peripheral ghrelin production. Interestingly, although stress loading increased feeding in female mice, plasma ghrelin levels were not increased, and upregulation of NPY/AgRP gene expression was not observed in the hypothalamus. Conversely, mRNA levels of the ghrelin precursor preproghrelin were significantly upregulated in the hypothalamus in stress-loaded female mice. Thus, in female mice, stress can increase ghrelin in the brain and directly promote feeding behavior. Alternatively, this may suggest the hypothesis that the mechanism of hyperphagia due to stress has different pathways in males and females. Thus, further verification is needed.

## 5. Stress-Induced Anorexia in Aged Subjects

### 5.1. Aging and Feeding

Currently, aging is a difficult challenge faced by developed countries [80,81]. Anorexia due to aging is a common condition in older people and occurs in about 20% of the older population [82]. Anorexia due to aging is defined as an age-related loss of appetite and food intake. It is also observed in healthy older adults without illnesses, and occurs even in those with an adequate food supply [83,84]. Loss of appetite can lead to protein–energy malnutrition and weight loss. In older adults, these are associated with several health conditions, including increased mortality [85]. The main causes of loss of appetite in this population, for example, the social environment and background in isolation from family, should be comprehensively evaluated. However, in terms of physiological aspects, the accepted concept states that energy consumption and supply demands decrease with age [86]. Additionally, abnormalities in the appetite-promoting or inhibitory satiety signals that regulate feeding may occur. For example, appetite-suppressing signals predominate in healthy older subjects more than in younger subjects, contributing to sustained satiety and hunger suppression [87].

Leptin and CCK, which are appetite-suppressing hormones, are also affected by aging. Animal studies have shown that centrally administered leptin induces anorexia and reduces fever effects in obese older rats [88]. However, clinical evidence on peripheral leptin levels in the elderly population varies. Although one study reported no difference in leptin levels among older patients with or without anorexia [82], another reported that leptin levels increased in middle-aged women and decreased in older women [89]. Additionally, leptin reportedly improves the satiety effect of cholecystokinin octapeptide (CCK-8) in aged animals [90]. This effect has also been observed in older individuals [91,92]. CCK levels increase with age in both animals [93] and humans [94]. Moreover, increased feeding behavior due to exogenous ghrelin administration is absent in aged mice compared with younger ones, and these mice also lack diurnal variation in peripheral ghrelin levels [95]. In humans, circulating ghrelin levels are lower in older adults than in young adults [96], but some studies have reported no difference [97]. Older individuals also have lower acylated ghrelin levels than deacylated des-acyl ghrelin levels [98]. Acylated ghrelin has a short half-life and is rapidly deacylated in circulation, leading to the formation of inactive des-acyl ghrelin. The difficulty of measuring ghrelin in clinical practice may explain the differences in these findings regarding ghrelin. Therefore, after blood collection, the inactivation of an acylated ghrelin-degrading enzyme is required as soon as possible within a predetermined time frame (acylated ghrelin has a half-life of about 10 min).

### 5.2. Aging and Stress

Compared with younger adults, older people have different biological reactions due to stressors. In older people, physical degeneration and illnesses accompanying aging may amplify the stress response [99]. As a result, anxiety and depression in this population are qualitatively different from those experienced by younger people, and disorders commonly resultant from aging, such as apathy, cognitive impairment, and sleep disorders, may overlap [81,100]. These disorders may indicate that the combination of aging and stress makes central functions more vulnerable.

For recovery from stress, glucocorticoids induced by HPA axis activation suppress and inhibit the production of CRF, AVP, and ACTH. The secretion of the most important endogenous glucocorticoid in the HPA axis has a constant diurnal rhythm, increases after waking up, and then slowly decreases in the evening [101,102]. Therefore, examining the diurnal variation of cortisol helps assess HPA axis activation. In rodents, some factors, including old age and recurrent stress [103], reduced glucocorticoid receptor (GR) reactivity [104,105]. When this occurs, the negative feedback on the HPA axis is reduced, resulting in the maintenance of high cortisol levels. Therefore, HPA axis dysregulation occurs in older adults, indicating that the HPA axis is vulnerable. Alterations in morning cortisol levels or the measurement of a cortisol-awakening response (CAR) are good indicators of HPA axis vulnerability in humans [106], and are flatter from morning to evening. A cortisol gradient has also been reported [107]. Almeida et al. [108] reported that older men had considerably higher CAR compared with younger men, whereas the effect of age in women was insignificant. This result also suggests that there are sex differences in age-related abnormal cortisol secretion.

Abnormal cortisol secretion in older adults can have serious clinical consequences. For example, overactivation of the HPA axis and hypersecretion of glucocorticoids can cause atrophy of the dendrites of hippocampal neurons, which can impair learning and memory functions, decision making, and emotional responses [109]. It has also been reported that glucocorticoids play a causal role in aging and age-related disorders [110] and speculated that the risk of developing neuropsychiatric disorders with aging increases. Persistent glucocorticoid elevation may also be associated with decreased reactivity in the GR on the HPA axis. Decreased levels and binding of the GR in the hippocampus and prefrontal cortex (PFC) have been demonstrated previously in aged animals [104,105]. For example, impaired feedback due to glucocorticoid inhibition impairs the GR in the hippocampus, PFC, and PVN in aged rats. Furthermore, it is associated with the inhibition of the GR translocation into the nucleus [104,105]. Thus, aging may cause a decline in GR function in the hippocampus, PFC, and PVN. In contrast, chronic stress and treatment with CORT do not cause cell death in the hippocampus of several animals, including humans [111,112]. These contradictory results are still being discussed.

### 5.3. Aging, Stress, and Feeding

There are few studies on stress and feeding in older adults. So far, psychological stress models in aged mice, such as novel environmental stress, have been used to demonstrate an increase in blood CORT levels and a decrease in feeding as compared with young mice [113]. Interestingly, in aged mice subjected to novel environmental stress loading, a decrease in the bout number was noted, as was a consistent decrease in meal amount and size. In normal-aged mice, one bout is originally small when feeding, but the number of bouts increases to maintain the daily feeding amount. It is assumed that, compared with young mice, older mice find it difficult to bite and eat large amounts of food at one time. In contrast, stress loading on aged mice reduces the increase in bout numbers, which is a typical feeding pattern. Thus, stress loading reduces food intake in aged mice and alters the feeding pattern itself. In studies on water-avoidance stress models using aged mice, there was a decrease in dark-term locomotor activity and an alteration in feeding behavior [19].

Glucocorticoids promote appetite in the short term [114,115,116,117]. However, the effect of chronic glucocorticoid exposure, which is a characteristic of stress loading in older adults, on appetite has not been fully investigated. High circulating ACTH levels reduce flavor cravings, and high cortisol levels reduce postprandial satisfaction and satiety in patients with Cushing’s syndrome. However, there seems to be no difference in hunger, fullness, and satisfaction between the fasting and fed conditions [118]. Fasting and postprandial circulating ghrelin levels are increased in those with Cushing’s syndrome because glucocorticoids stimulate the expression of ghrelin and its receptor [119,120,121]. Furthermore, no relationship was found between fasting or postprandial ghrelin levels and appetite or thirst scores. These findings may evince that chronic glucocorticoid exposure is a part of ghrelin-mediated appetite abnormalities in older adults.

The persistent reduction in feeding behavior during acute stress loading in aged mice appears to be mediated by complex networks in the periphery and brain. One may involve increased sensitivity of serotonin 2C receptors (5-HT_2C_R) in the brain [113,122,123]. 5-HT_2C_R is a receptor primarily involved in anxiogenic and feeding control [124,125], and intraperitoneal or intracerebroventricular administration of the 5-HT_2C_R stimulant m-chlorophenylpiperazine (mCPP) reduces food intake [43,113] while simultaneously reducing peripheral ghrelin secretion [43]. As previously reported, the intraperitoneal administration of mCPP in mice reduces food intake and appears to have a stronger effect in aged mice than in younger ones. Conclusively, the administration of 5-HT_2C_R antagonists almost reverses the persistent decrease in feeding and the increase in the HPA axis due to stress loading in aged mice [113,122]. Although the mechanism is not completely clear, 5-HT_2C_R mRNA expression in the hypothalamus, which receives peripheral hunger signals, is significantly enhanced by stress loading in aged mice [113]. The number of 5-HT_2C_R-expressing cells also increases in aged mice. The increased production of 5-HT_2C_R in the brain may play an important role in stress-bearing vulnerability in aged mice. Because ghrelin kinetics are abnormal in normal older mice [95], stress loading on older adults is expected to cause complex biological reactions. It is speculated that aged mice with high sensitivity to 5-HT_2C_R have a more pronounced decrease in ghrelin than young mice, but appropriate comparative studies investigating this have not been conducted so far. Further stress loading should cause highly complex biological reactions. On the other hand, chronic stress-induced binge eating observed in young mice was not seen in aged mice. Changes in stress response with aging appear to differ between acute and chronic stresses.

There are few studies on sex differences in older adults under stress load. We suggest that, compared with aged female mice, aged male mice are extremely vulnerable to stress [122,123]. The results of novel environmental stress loading revealed a sustained decrease in food intake and hyperactivity of the HPA axis in aged male mice. However, the exposure of aged female mice to stress on a similar schedule results in no sustained response. This may be related to clinical findings demonstrating that HPA axis abnormalities with age are absent in women [108]. Additionally, stress-loaded aged female mice have lower neural activity in the amygdala and PVN than males, and do not show overactivation of the HPA axis; further, the intraperitoneal administration of mCPP does not show as much responsiveness in female mice as in male mice [122]. Compared with young mice, aged male mice demonstrate the overexpression of 5-HT_2C_R, but female mice do not demonstrate the hyperactivity of 5-HT_2C_R-positive neurons with aging. The decrease in peripheral ghrelin levels in aged male mice is not observed in female mice. Compared with aged female mice, male mice tend to have higher mRNA expression of ERα, ERβ, and aromatase in the hypothalamus under normal conditions. Furthermore, it has been demonstrated that male mice are more sensitive to stimulants of the ERα rather than ERβ, but females are less sensitive than males to the same [122]. Further studies are needed to determine the direct relationship between ERα activation and reduced feeding through 5-HT_2C_R activation.

## 6. Conclusions

**Figure 1:**
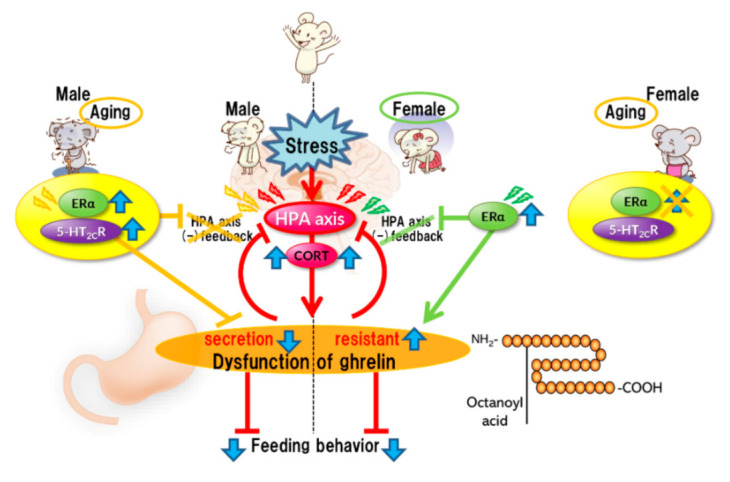
Acute stress.

**Figure 2:**
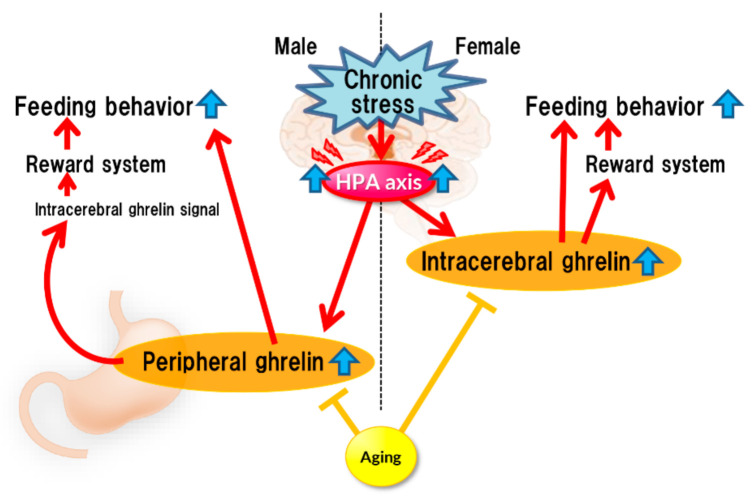
Chronic stress.

Stress affects eating behavior, a basic but important activity. A complex network of appetite-related peptides controls appetite. When multiple factors, such as disease complications, sex differences, and aging, are involved, appetite control becomes more complex. Thus, at present, it remains challenging to completely understand appetite control. For example, the orexigenic peptide, ghrelin, mediates stress-induced eating disorders by abnormal secretion and signal transduction. Disturbances in ghrelin signals are also influenced by aging and sex. Sex differences also regulate ghrelin responsiveness, and aging triggers a persistent stress response (Figure 1). However, overeating due to chronic stress does not occur with aging (Figure 2). Further research on each reactivity according to each type of stress is warranted.

Acute stress rapidly activates the HPA axis, inducing the negative feedback system for stress. Simultaneously, stress causes abnormal ghrelin secretion, consequently leading to anorexia. It is considered that the mechanism of ghrelin dysfunction varies depending on the sex. In females, ERα causes dysregulation of the HPA axis negative feedback. ERα activation may be involved in the induction of ghrelin resistance. Aging results in the aberrant activation of serotonin 2C receptors under stress in male mice, leading to the disruption of the negative feedback system of the HPA axis and amplification of stress-induced biological responses. Aberrant activation of serotonin 2C receptors in the central nervous system is unlikely to occur in aged female mice.

Chronic stress continuously activates the HPA axis, which enhances ghrelin secretion (or reaction enhancement) in the brain, thereby stimulating the reward system, which could lead to increased feeding. Such enhancement may not occur in aged mice.

## Data Availability

Not applicable.
